# Research on the mathematical model and design of a three loop controller for the oscillating scanning system of a laser line scanning galvanometer

**DOI:** 10.1038/s41598-023-46245-2

**Published:** 2023-11-01

**Authors:** Xiaoyan Du, Jianying Li, Wenjie Zhao

**Affiliations:** 1 School of Mechanical and Power Engineering, Harbin University of Science and Technology, Harbin, 150080 China; 2 Key Laboratory of Advanced Manufacturing and Intelligent Technology, Ministry of Education, Harbin, 150080 China

**Keywords:** Electrical and electronic engineering, Mechanical engineering

## Abstract

The scanning vibration mirror system drives the scanning mirror fixed to it through the oscillation of the motor shaft, so as to control the reflected light to form a dynamic variable light path. The vibration mirror scanning system has higher controllability than the fixed optical path system and has been widely used.In this dissertation, after establishing the models of the core components of the scanning vibration mirror system, the mathematical model of the whole system is established.On this basis, simulation and theoretical analysis show that the system has some shortcomings, such as small bandwidth, low dynamic tracking accuracy, and the comprehensive dynamic performance of the system is easily affected by the input of external interference branches. A series correction controller and three closed-loop controller are designed for the above problems, respectively, and the control effects of the two controllers on the scanning vibration mirror system are studied through simulation experiments. By comparing the output response results of the system under the action of sinusoidal signals of different frequencies, it can be seen that the comprehensive effect of the three closed-loop controllers is better. Under the action of step signals, the overshoot of the three closed-loop correction controller correction system is 21.5% higher than that of the series controller correction system, the adjustment time is 82.7% less, and the steady-state error is significantly smaller. Therefore, it indicates that the three closed-loop correction system has good rapidity and steady-state accuracy.

## Introduction

With the continuous development of precision opto-electromechanical technology, laser scanning technology has also been widely used in military, aerospace, laser engraving and medical treatment^1, 2^, Among them, vibration mirror scanning has attracted much attention because of its small size, high speed, high precision, flexible operation and change of optical path.The vibration mirror system is mainly composed of motor control board, torque motor, scanning lens and encoder.Among them, the torque motor integrates the scanning lens, the position encoder and the control board, and can meet the better dynamic performance and adapt to the changes and disturbances of the environment^3^. The vibration mirror motor control system is a classic servo control system, which can realize the characteristics of high precision and high frequency operation of the vibration mirror.

At present, scholars at home and abroad have also done a lot of research on vibration mirrors to achieve high-frequency work and high-precision work. Li et al.^4^, in order to improve the control accuracy and anti-interference performance of the vibration mirror system, a fuzzy adaptive PID control strategy was designed by combining PID control and fuzzy control. Compared with the traditional PID control strategy, this strategy has better dynamic and static performance and anti-interference ability. Zaeh et al.^5^, used adaptive inverse control to improve the dynamic performance of the vibration mirror, compensated the dynamic model by considering the working characteristics of the vibration mirror mirror, and verified the feasibility of the method through experiments. Optical errors are reduced by about 95% compared to conventional galvo scanning systems. Tomohiko et al.^6^, designed a real-time motion blur compensation system for the high-speed one-dimensional motion between the camera and the target. The system has no sensor and is composed of a vibration mirror and a high-speed camera. The control of the vibration mirror motor is realized through the repeated oscillating motion of the high-speed camera, which can feedback the angular velocity of the vibration mirror within 10 ms, and can capture moving images with a speed of up to 30 km/h. When the vibration mirror works at high speed, it will generate a high temperature, which will cause thermal demagnetization, and the torque generated by the vibration mirror will decrease with the increase of temperature. Based on the above problems, Matsuka et al.^7^, developed a thermal demagnetization compensation method, which estimates the temperature of the magnet based on the detected current and performs torque compensation. Maeda et al.^8^, proposed a deadbeat control method with adaptive feedforward compensation. This method can reduce the resonant frequency fluctuations caused by temperature changes, product aging and other problems, and can improve the robustness of the vibration mirror system. In nonlinear optimization, the obtained parameters depend on the initial conditions, while the initial point based on trial and error in practical work requires a large amount of work. Based on the above problems, Kuroda et al.^9^, used the system optimization technology with simple initial parameters to eliminate the problem of feedback controller design of series structure. Han et al.^10^, in order to achieve high-speed, linear and accurate scanning of the vibration mirror, the method of time delay approximation and zero-phase approximation is used to design an iterative learning control strategy. The experimental verification shows that the root mean square error of iterative learning can be reduced by 73 times compared with the controller of the commercial vibration mirror system. In order to solve the problems of poor anti-interference ability and difficulty in mass production in the vibration mirror scanning control system, Cao et al.^11^, designed a high-speed digital vibration mirror control scheme. Using digital notch filter to suppress mechanical resonance, a digital vibration mirror control system based on DSP is developed. The experiment shows that the bandwidth of the system can reach 300 Hz, and the operation is stable and reliable.Chou et al.^12^, in order to suppress the vibration of the vibration mirror and improve the accuracy and stability of the laser vibration mirror scanning system, they developed a laser vibration mirror vibration measurement, analysis and suppression system. The system includes a computer, an intelligent motion control platform, a servo drive circuit, and a drive module. Experiments show that the system can effectively reduce system vibration and improve the accuracy and stability of vibration mirror scanning. In order to solve the problem that the vibration mirror takes a long time to reach steady state when it starts and stops and switches the frequency domain and amplitude, Barrett et al.^13^, proposed a new open-loop control strategy.The ability to excite system modes through complete high-speed control is called modal engineering, and this strategy can be used to quickly switch the speed position, frequency domain and swing angle. Yuan et al.^14^, in order to improve system compatibility, portability and network management, proposed an FPGA-based Ethernet laser vibration mirror controller.The controller can accurately control the swing angle of the vibration mirror, flexibly support the heterogeneous host computer environment, and solve the problem of software and hardware compatibility for data transmission based on the traditional PCL interface vibration mirror control card. Barrett et al.^15^, used a feedforward control compensation method for the impulse response of the vibration mirror motion to improve the motion accuracy of the vibration mirror motor. In order to reduce the noise of the angle sensor to achieve higher quality motion, Ito et al.^16^, used an inverse iterative control (IIC) algorithm to make the vibration mirror scan at a swing angle of ± 10° at 20 Hz, and successfully use noise reduction to Residual tracking error is reduced by 41%. Sasago et al.^17^, used a new technique for monitoring and compensating the scanning path of the galvo mirror using a position-sensitive detector to compensate for the manufacturing error of the fiber optic scanner. Experiments show that by properly controlling the amplitude and phase difference of the applied voltage, the beam can be linearly scanned at the resonance frequency of about 10 kHz. Wenyuan et al.^18^, proposed a high-precision position control method based on Active Disturbance Rejection Control (ADRC) for the vibration mirror scanner system. This method has a simple control structure and a small amount of calculation, which can ensure the high dynamic performance and high precision of the system position control.Wang et al.^19^, used the calibration method of the double checkerboard calibration plate to calibrate the vibration mirror scanning system, and established the error model of the system and theoretically analyzed the measurement error during the scanning process. Finally, a compensation method based on look-up table method is proposed for the angle error of the vibration mirror. It is proved by experiments that this method can significantly improve the measurement accuracy and robustness of the system. Wang et al.^20^, designed a composite control method based on feedforward and feedback; this control method improves the tracking performance of the vibration mirror system through the zero phase difference tracking controller (ZPETC). The additive decomposition outputter is used to suppress the disturbance caused by the uncertainty of the system, and then the differential evolution algorithm is used to design a PID controller without overshoot to compensate the residual error. The simulation results show that the combined controller can effectively reduce the influence of system uncertainty in the tracking process and ensure that the vibration mirror system has good tracking performance. Wang et al.^21^, on the basis of establishing the mathematical model of the vibration mirror system, studied the control model of PID plus feedforward control, and through simulation and experiments, proved that PID plus feedforward control can greatly improve the rapidity of marking response and precision. Quan^22^, established a mathematical model of the scanning mechanism and position feedback optical path on the basis of the structure and working principle of the laser scanning vibration mirror, and proposed a laser scanning vibration mirror control based on double closed-loop control on the basis of the system mathematical model. The algorithm effectively improves the motion accuracy of the vibration mirror. Chen et al.^23^, in order to eliminate the nonlinear scanning error of vibration mirror scanning system in laser rapid prototyping machine and improve the scanning accuracy of the system, a method of correcting the error of vibration mirror scanning system by using neural network is proposed. The experimental results show that the Elman neural network is used to construct the error compensation surface of the vibration mirror scanning system, and the dynamic error correction of the vibration mirror scanning system can significantly improve the scanning accuracy of the rapid prototyping machine.

Domestic and foreign scholars mainly focus on the research of intelligent control strategies and artificial neural network control to improve the dynamic performance of galvanometer systems, especially in improving signal-to-noise ratio and reducing thermal magnetic effects. Intelligent control strategies are designed for highly complex nonlinear systems and systems with uncertain mathematical models, and can be better handled through technologies such as deep learning. However, these methods require complex model training and parameter adjustment, as well as expensive hardware and computational resources. Given that the galvanometer structure is relatively simple and does not belong to highly complex nonlinear systems, the three closed-loop control strategy proposed in this manuscript is based on classical control theory to meet the requirements of the galvanometer system. This strategy does not require complex model training and is suitable for real-time applications and embedded systems. Its parameters and operation mode can usually be clearly explained and understood, which helps engineers and operators to adjust and optimize control systems more easily. In addition, this control strategy does not require expensive hardware and computing resources, and has a lower cost advantage. By adopting this three closed-loop control strategy based on classical control theory, it can meet the performance requirements of the galvanometer system while reducing the complexity and cost of the system, making it more suitable for practical engineering applications. Due to the high operating frequency of the vibration mirror system, the research on dynamic performance is mainly carried out from two perspectives: rapidity and accuracy. For some mechanisms that implement laser surface scanning, such as laser cleaning^24^, laser sterilization^25^ and other mechanisms, the requirements for the phase angle lag of the vibration mirror during the working process are relatively low. Therefore, it is of great significance to reduce the dynamic and static errors of the vibration mirror system by sacrificing the technical indicators of phase angle lag. In this paper, each component of the vibration mirror system is firstly analyzed and its mathematical model is established, and the frequency domain characteristics of the system are analyzed by using Matlab software. According to the system characteristics, series correction and three closed-loop control strategies (current loop, velocity loop and position loop) are designed. Finally, the corrected system is analyzed in frequency domain and time domain through Simulink software. The results show that the controller can increase the bandwidth of the system, and has good dynamic performance such as high tracking accuracy under high frequency working conditions.

## Mathematical model establishment of scanning galvo system

### Composition of scanning vibration mirror system

**Figure 1:**
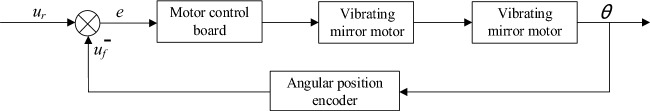
The composition block diagram of the vibration mirror swing-sweep system.

The scanning vibration mirror system consists of a vibration mirror motor, an angular displacement encoder, a signal generator, and a scanning lens. The frequency, rotation angle and angular velocity of the vibration mirror when the vibration mirror is driven by the motor are the main parameters of the scanning vibration mirror system. The vibration mirror motor is a precision motor with limited angular displacement. It drives the scanning mirror fixed to it through the swing of the motor shaft, thereby controlling the reflected light to form a dynamic variable optical path. Figure 1 is a block diagram of the scanning vibration mirror system^26^. The galvanometer swing scanning system is a closed-loop system. The galvanometer swing scanning system receives the input signal ur, and the galvanometer will follow the amplitude and frequency of the input signal ur to produce a swing effect. The angle position information of the galvanometer is converted into a feedback position signal uf through an angle position encoder. The angle error e is the difference between the input signal ur and the angle position feedback signal uf. Therefore, how to quickly eliminate and suppress the range of angle errors is a key factor in improving the dynamic performance of the mirror swing scanning system.

### Mathematical model establishment of vibration mirror system

#### Mathematical model establishment of angular displacement encoder

The pulse signals output by the photoelectric incremental encoder are A-type, B-type and Z-type signals. The A-type and B-type signals can calculate the angular velocity and angle of the vibration mirror, and the Z-type signal is a zero-bit signal. According to the output pulse signal A and pulse signal B, the motor rotation state is obtained, and the output response speed and output angle can be obtained according to the response frequency, resolution and response time. The encoder speed is measured by the M method, that is, the speed information is calculated according to the number of output pulses in a unit time, and the speed calculation equation is shown in Eq. (1).

1 $$n = \frac{{2\pi m_{1} }}{{NT_{cu} }}$$

Its angle $\theta_{\alpha }$ is calculated by Eq. (2):

2 $$\theta_{\alpha } = P \times n$$

In the Eq. (2), *P* is the pulse equivalent, $P = {{360} \mathord{\left/ {\vphantom {{360} N}} \right. \kern-0pt} N}$, *N* is the resolution, that is, the number of pulses per revolution, *n* is the number of pulses generated by the forward or reverse signal A or B, $T_{cu}$ is the detection time, and $m_{1}$ is the number of pulses in the detection time.

#### Mathematical model of vibration mirror motor

The equation for calculating the rotational torque of the vibration mirror motor spindle is:

3 $$T = BLNID - 2\pi 10^{ - 7} nHN^{2} I^{2} \frac{D}{h}$$

In the Eq. (3), *T*(*N·m*) is the electromagnetic torque generated by the coil current of the vibration mirror motor, *B*(*T*) is the magnetic induction intensity generated by the permanent magnet in the space gap, *H*(*m*) is the length of the rotor, *D*(*m*) is the diameter of the rotor, *h*(*m*) is the space gap distance, *N* is the number of turns of the coil, *I*(*A*) is the current of the coil, and n is the ratio of the maximum rotor angle to the actual rotation angle. In practical work, the influence of the nonlinear term in Eq. (3) is much smaller than that of the linear term, so the following relationship can be obtained after simplification:

4 $$T = BLNID$$

In the Eq. (4), *BLND* is the inherent property of the vibration mirror motor, and $K_{T} = BLND$ can be set, so the above equation can be simplified as:

5 $$T = K_{T} I$$

According to Eq. (5), it can be known that the electromagnetic torque generated by the coil current of the vibration mirror motor is proportional to the current. The rotational balance torque equation of the vibration mirror motor is:

6 $$T - T_{c} = J\frac{{d^{2} \theta }}{{dt^{2} }} + \mu \frac{d\theta }{{dt}}$$

$T$ is the electromagnetic torque generated by the coil current of the vibration mirror motor; $J$ is the rotational inertia of the vibration mirror motor rotor, $\theta$ is the deflection angle of the vibration mirror motor rotor, $\mu$ is the viscous friction damping coefficient inside the vibration mirror motor, $T_{c}$ is the vibration mirror motor Total load torque on the shaft.

The armature balance equations of the motor is^27^:

7 $$u = RI + L\frac{dI}{{dt}} + E_{b}$$

8 $$E_{b} = K_{b} \frac{d\theta }{{dt}}$$

where *R* is the armature resistance and $K_{b}$ is the back electromotive force coefficient of the motor.

When the control voltage *u* is applied to both ends of the armature winding, the armature current *I* is generated, and the electromagnetic torque *T* is obtained immediately, which drives the armature to overcome the resistance torque, drives the load to rotate, and generates a back electromotive force *E*_b_ at both ends of the armature.

Substituting the above Eq. (5) into Eq. (6) and Eq. (7) into (8), the following relational equations can be obtained:

9 $$IK_{T} - T_{c} = J_{z} \frac{{d^{2} \theta }}{{dt^{2} }} + \mu \frac{d\theta }{{dt}}$$

10 $$u = RI + L\frac{dI}{{dt}} + K_{b} \frac{d\theta }{{dt}}$$

Further simplification results in the following form:

11 $$LJ_{z} \frac{{d^{3} \theta }}{{dt^{3} }} + (L\mu + RJ)\frac{{d^{2} \theta }}{{dt^{2} }} + (R\mu + K_{T} K_{b} )\frac{d\theta }{{dt}} = K_{T} u - L\frac{dT}{{dt}} - RT$$

The Laplace transform of (9) and (10) can be obtained:

12 $$IK_{T} - T_{c} = J_{z} s^{2} \theta + \mu s\theta$$

13 $$u = RI + LIs + K_{b} \theta s$$

**Figure 2:**
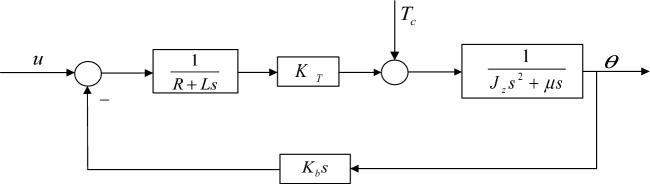
Block diagram model of the vibration mirror motor system.

In the equations, R is the armature resistance, $K_{b}$ is the motor back EMF coefficient, and L is the armature circuit inductance. Simplify according to the above Eqs. (12) and (13), the system block diagram can be obtained as shown in Fig. 2.

The mathematical model of the transfer function of the system can be obtained, and its equation is shown in Eq. (14):

14 $$LJ_{z} \theta s^{3} + (L\mu + RJ)\theta s^{2} + (R\mu + K_{T} K_{b} )\theta s = K_{T} u - LTs - RT$$

When the interference* T* = 0, the above Eq. (15) can be simplified to the following form:

15 $$\begin{gathered} \\_{b} \\ \\ \\ \end{gathered}$$

When *U* = 0 is input, Eq. (14) can be simplified to the following form:

16 $$\frac{\Theta }{{T_{c} }} = \frac{{ - \left( {Ls + R} \right)}}{{LJ_{z} s^{3} + (L\mu + RJ_{z} )s^{2} + (R\mu + K_{T} K_{b} )s}}$$

Therefore, according to Eqs. (15) and (16), it can be known that under the combined action of the input signal *u* and the interference *T*, the angle is as follows:

17 $$\Theta = \frac{{K_{T} U - T_{c} \left( {Ls + R} \right)}}{{LJ_{z} s^{3} + (L\mu + RJ_{z} )s^{2} + (R\mu + K_{T} K_{b} )s}}$$

The basic parameters of the known vibration mirror motor are shown in Table 1:

**Table 1:** Galvo system parameters.

Parameter	Numerical value
Coil resistance *R*	2.55 Ω
moment coefficient *K*_*T*_	0.023 N m/A
Moment of inertia of vibration mirror system *J*	1.254 × 10^–7^ kg m^2^
Reverse electromotive force coefficient *K*_*b*_	3.82 × 10^–4^ V s/°
Armature inductance *L*	1.1 × 10^–4^ H

### Analysis of motor moment of inertia and load torque

The load of the vibration mirror motor is the vibration mirror lens, and the load torque is determined by the moment of inertia of the vibration mirror system. The moment of inertia mainly includes the moment of inertia of the vibration mirror lens, the moment of inertia of the rotor and the equivalent moment of inertia of other connected components. Therefore, the moment of inertia of the system is shown in Eq. (18).

18 $$J = J_{p} + J_{z} + J_{o}$$

In the Eq. (18), $J_{p}$ is the moment of inertia of the lens, $J_{z}$ is the moment of inertia of the rotor, and $J_{o}$ is the moment of inertia of the connecting part.

It can be known from Eq. (18) that the load torque of the system is shown in Eq. (19).

19 $$T_{j} = J\omega$$

In the Eq. (19), $\omega$ is the angular velocity of the vibration mirror.

### Frequency domain analysis of galvo system

According to Eq. (17), it can be seen that the mathematical model of the vibration mirror motor belongs to the third-order system mathematical model. Since the friction damping coefficient of the vibration mirror is not given, when the friction damping is not considered, and the moment of inertia and load torque are shown in Eqs. (18) and (19), respectively, Eq. (20) can be obtained:

20 $$\Theta = \frac{{K_{T} U - T_{c} \left( {Ls + R} \right)}}{{LJs^{3} + RJs^{2} + K_{T} K_{b} s}}$$

Due to the small load torque and inductance of the system, when the external load disturbance torque is ignored, and Table 1 is put into Eq. (20), the output equation of the system can be obtained as shown in Eq. (21).

21 $$\frac{\Theta }{U} = \frac{{K_{T} }}{{LJs^{3} + RJs^{2} + K_{T} K_{b} s}} = \frac{0.023}{{8.25 \times 10^{ - 12} s^{3} + 3.198 \times 10^{ - 7} s^{2} + 8.78 \times 10^{6} s}}$$

Since the value of *LJ*($LJ = 8.25 \times 10^{ - 12}$)is small, Eq. (21) can be further simplified to obtain Eq. (22).

22 $$\frac{\Theta }{U} = \frac{{K_{T} }}{{RJs^{2} + K_{T} K_{b} s}} = \frac{71917.95}{{s^{2} + 27.455s}}$$

**Figure 3:**
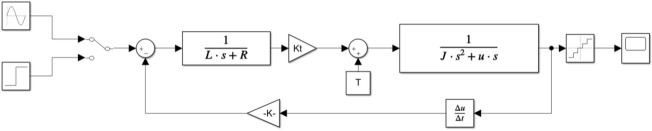
Simulink model of vibration mirror system.

**Figure 4:**
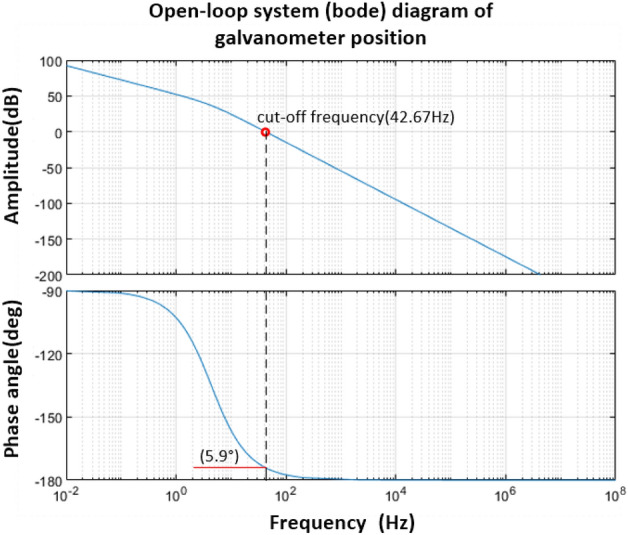
Bode diagram of open-loop system of vibration mirror motor without friction damping.

According to the block diagram and Eq. (22) and the quantizer model of the incremental encoder number, the Simulink simulation model can be obtained, as shown in Fig. 3:

When the vibration mirror system does not consider the influence of the viscous friction coefficient, according to Eq. (22), it can be known that the Bode diagram of the open-loop system is shown in Fig. 4.

It can be seen from Fig. 4 that when the viscous friction damping coefficient is ignored, the cut-off frequency $\omega_{c} = 42.67\;{\text{Hz}}$, the phase angle margin is 5.9°, and the amplitude margin is greater than 10 dB. It can be seen that the system meets the stability conditions, but because the phase angle margin is too small, so the system stability is low.

In practical systems, the effect of viscous friction damping cannot be ignored. According to the characteristics of the motor during operation, taking the viscous friction coefficient $\mu = 0.8 \times 10^{ - 4}$, the open-loop transfer function of the system is shown in Eq. (23):

23 $$\frac{\Theta }{U} = \frac{{K_{T} }}{{LJs^{3} + (L\mu + RJ)s^{2} + (R\mu + K_{T} K_{b} )s}} = \frac{{2.788 \times 10^{9} }}{{s^{3} + 1.881 \times 10^{4} s^{2} + 2.713 \times 10^{7} s}}$$

Equation (23) can be simplified as shown in Eq. (24):

24 $$\frac{\Theta }{U} = \frac{{\frac{{K_{T} }}{{(R\mu + K_{T} K_{b} )}}}}{{s\left( {\frac{(L\mu + RJ)}{{(R\mu + K_{T} K_{b} )}}s + 1} \right)}} = \frac{K}{{s\left( {T_{m} s + 1} \right)}}$$

Then the system damping ratio can be obtained from Eq. (25):

25 $$\varsigma = \frac{1}{{2\sqrt {T_{m} K} }} = \frac{1}{{2\sqrt {\frac{(L\mu + RJ)}{{(R\mu + K_{T} K_{b} )}} \cdot \frac{{K_{T} }}{{(R\mu + K_{T} K_{b} )}}} }} = 1.224$$

**Figure 5:**
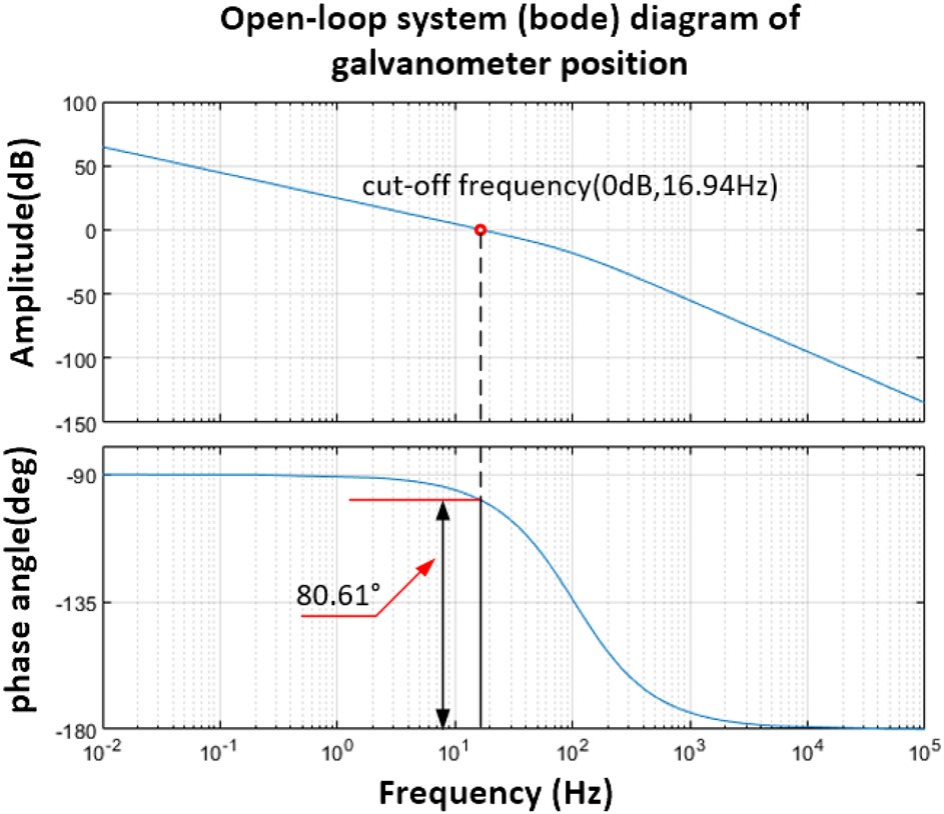
Bode diagram of open-loop system of vibration mirror motor considering friction damping.

It can be seen that under this working condition, the system belongs to the over-damped state, and the open-loop Bode diagram of the vibration mirror system can be obtained by considering the viscous friction damping coefficient, as shown in Fig. 5.

From the analysis of Fig. 5, it can be seen that the cut-off frequency of the system is $\omega_{c} = 16.94\;{\text{Hz}}$, and the corresponding phase angle margin is 80.61. It can be seen that the system meets the stability conditions, but its open-loop cut-off frequency is small. Compared with Fig. 4, it can be seen that the viscous friction damping will have a large difference in the frequency domain characteristics of the oscillating vibration mirror motor system. Therefore, in the subsequent calibration of the vibration mirror system and controller design, the viscous friction damping should be considered The influence of factors is more in line with the actual situation.

**Figure 6:**
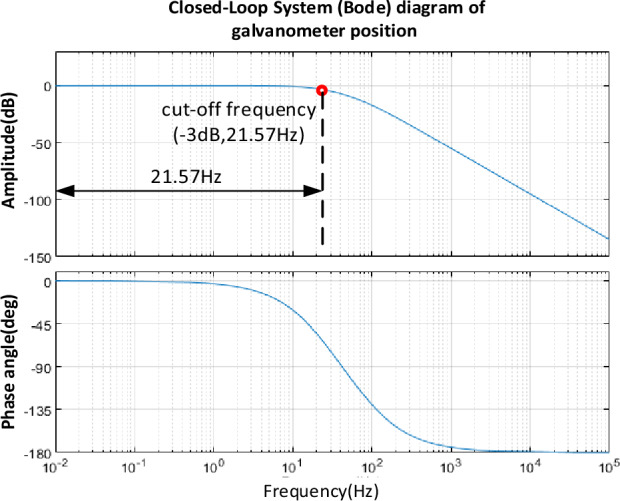
Bode diagram of closed-loop system of vibration mirror motor considering friction damping.

To verify the accuracy of the mathematical model of the designed galvanometer system, the closed-loop frequency domain characteristic curve of the system was visualized, as shown in Fig. 6. In this curve, the system's − 3 dB cutoff frequency is 21.57 Hz. At the same time, we also referred to the research in reference^28^, which identified the galvanometer system and obtained a closed-loop frequency curve. According to analysis, the − 3 dB cutoff frequency of the galvanometer system in the literature is 35.92 Hz. It is worth noting that the phase angle curves of the two almost completely coincide. Based on the above analysis process, it is shown that the established mathematical model is very accurate and has a high level of confidence. This result also proves the accuracy of the mathematical model in describing system behavior.

## Design of the controller

In order to make the vibration mirror achieve the characteristics of high frequency oscillation when it scans, it is necessary to design a new effective controller for it. According to the high-frequency motion and dynamic performance requirements of the actual high-speed ultraviolet laser scanning system as the design indicators, compared with the series correction, a new controller based on the three closed-loop correction strategy is designed for the system, and the dynamic characteristics of the system under the action of them are compared and analyzed.

### Design of series correction controller

The bandwidth of a system usually needs to be greater than a certain multiple of the system's operating frequency to ensure that the system has sufficient bandwidth near the operating frequency. Generally speaking, setting the system bandwidth to twice or more the system's operating frequency is a common practice. Considering that the working frequency of the system is around 100 Hz, and the series correction only uses position closed-loop method, the ability to suppress noise interference is relatively weak. If the system bandwidth is designed too large, it will introduce more noise interference. Therefore, considering the closed-loop form and characteristics of the system, the closed-loop bandwidth of the system is designed to be about twice the operating frequency of the system. Reference^29^ indicates that appropriate phase angle and amplitude margins can prevent the impact of component aging on performance in the system. To achieve satisfactory performance, the phase margin should be between [30, 60] degrees, and the amplitude margin should be greater than 6 dB. Therefore, this manuscript adopts a design parameter with a phase margin of 45° and an amplitude margin of 10 dB when designing an open-loop system. Due to the fact that the system bandwidth (closed-loop cutoff frequency) is usually slightly greater than the open-loop cutoff frequency, the cutoff frequency of the designed galvanometer open-loop system is $\omega_{c} \ge 150\;{\text{Hz}}$. Since the cut-off frequency of the original system is only $\omega_{c} = 24.09\;{\text{Hz}}$, it does not meet the requirements; at the same time, when $\omega_{c} = 150\;{\text{Hz}}$, its amplitude is − 23.6 dB, so the amplitude-frequency characteristic curve should be shifted upward. According to the above characteristics, the PD lead correction controller is designed for the system as a series correction link, and the form of the PD controller is set as follows.

26 $$G_{c} {(}s{)} = K\left( {{1} + Ts} \right)$$

According to the calculation,$K = 10.7072$, then, if the time constant $T = {1 \mathord{\left/ {\vphantom {1 {300}}} \right. \kern-0pt} {300}}\pi$, the PD correction link is as follows:

27 $$G_{c} (s) = \left( {10.7072 \times \left( {1 + \left( {1/300\pi } \right)s} \right)} \right)$$

Then the corrected open-loop transfer function of the system is:

28 $$H_{c} (s) = \frac{\Theta }{U} \cdot G_{c} (s) = \frac{721.832s + 680,335.236}{{s^{2} + 389.9s}}$$

**Figure 7:**
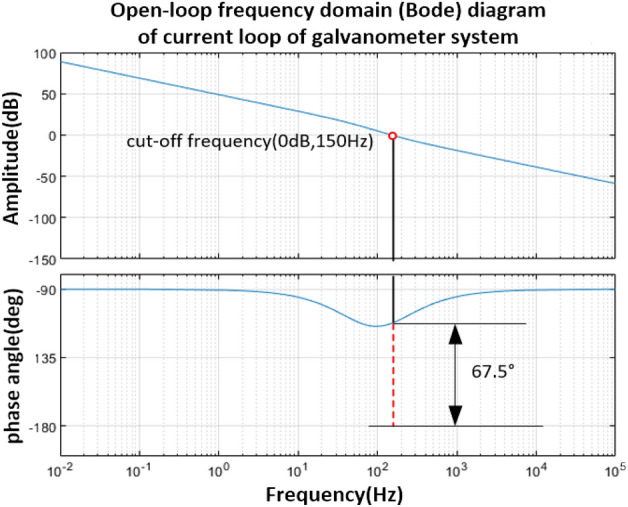
Bode diagram of open-loop system with series correction.

From Eq. (28), it can be obtained that after the series PD correction control, the open-loop frequency domain curve of the system is shown in Fig. 7.

**Figure 8:**
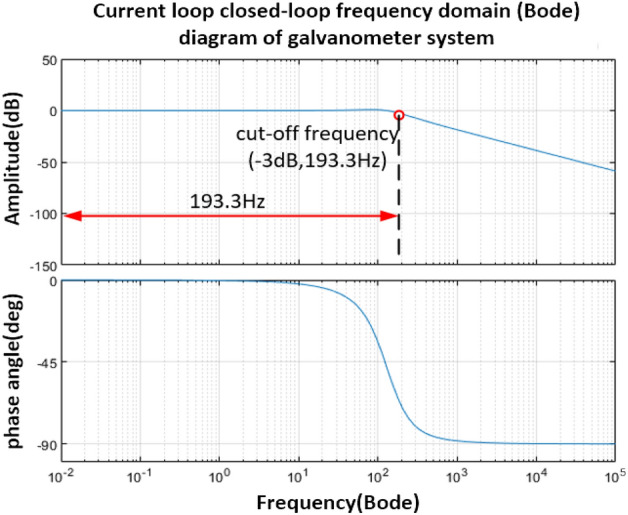
Bode diagram of closed-loop system after series correction.

As shown in Fig. 7, after the series PD correction control, the system cut-off frequency $\omega_{c} = 150\;{\text{Hz}}$, and the phase angle margin is 67.5°, and the amplitude margin meets the stability requirements. And further obtain its closed-loop Bode diagram as shown in Fig. 8, the bandwidth of the vibration mirror closed-loop control system is 193.3 Hz.

### Design and simulation of three closed-loop controller for vibration mirror system

**Figure 9:**
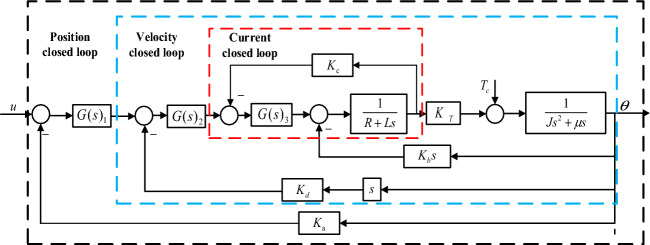
Block diagram of three closed-loop correction control of vibration mirror system.

After the series PD correction control of the system, the bandwidth of the vibration mirror closed-loop system is still relatively small, and the response speed is relatively slow, so it is difficult to meet the need of high-frequency scanning of vibration mirror swing scanning system. Therefore, it is necessary to further increase the bandwidth of vibration mirror system and keep the stability of the system. Although the desired characteristic method of series correction is easy to implement, its dynamic performance is poor. After analysis and comparison, a new controller is designed for the system according to the three closed-loop control strategies, as shown in Fig. 9. Among them, $G(s)_{1}$ is the position loop controller; $G(s)_{2}$ is the speed loop controller; $G(s)_{3}$ is the current loop controller, and $K_{a}$, $K_{d}$, and $K_{c}$ are the feedback coefficients of the corresponding links respectively.

#### Design of vibration mirror current loop controller

**Figure 10:**
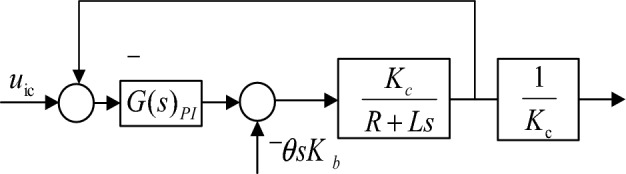
Block diagram of current closed-loop system.

The current loop unit is fed back to the input to form a local loop as shown in Fig. 10. Then, the frequency characteristics of the system are analyzed, and then the current loop controller meeting the requirements is designed.

As shown in Fig. 10, the input terminal is *u*_ic_, and $\theta sK_{b}$ is the external interference term. Since the system viscous friction coefficient $\mu = 5.19 \times 10^{ - 5}$ under actual working conditions, the electromagnetic time constant ${{T_{{\text{e}}} = L} \mathord{\left/ {\vphantom {{T_{{\text{e}}} = L} R}} \right. \kern-0pt} R} = 4.3137 \times 10^{ - 5}$.Among them, the motor time constant ${{T_{{\text{m}}} = RL} \mathord{\left/ {\vphantom {{T_{{\text{m}}} = RL} {\mu + K_{b} }}} \right. \kern-0pt} {\mu + K_{b} }}K_{T} = 5.405$, that is, $T_{e} < < T_{m}$, so the change of the current is much greater than the influence of the change of speed on the system.

And because $K_{b}$ is small, it can be regarded as a low-frequency noise effect.

Known inductance $L = 1.1 \times 10^{ - 4} H$, $R = 2.55\Omega$, feedback coefficient $K_{c} = 0.8$, its open-loop transfer function is as follows:

29 $$G{\prime}_{3} (s) = \frac{{K_{c} }}{Ls + R} = \frac{0.8}{{\left( {1.1 \times 10^{ - 4} } \right)s + 2.55}}$$

Since the phase angle of the UV laser swing-sweep device designed in this study is not the object of study, only the compensation gain is needed for the current loop controller. Therefore, the current loop controller is the PI control link, and its controller model is shown in Eq. (30).

30 $$G_{PI} = \frac{K}{Ts} + K$$

From Eq. (29), it can be seen that the current loop turning frequency $\omega_{{\text{j}}} = 23,180.34\;{\text{Hz}}$. In view of the operating frequency of around 100 Hz, it is necessary to ensure that the system has sufficient bandwidth near the operating frequency and comprehensively consider reducing the impact of high-frequency noise. Appropriate adjustments can be made within two orders of magnitude, and in combination with recommendation $\omega_{c} \ge {3000}\;{\text{Hz}}$ in reference^3^, the current loop bandwidth should be taken. Therefore, when $\omega_{c} = {3000}\;{\text{Hz}}$, then $L(\omega_{c} ) = - 12.29\;{\text{dB}}$, and the proportional coefficient *K* is solved as shown in Eq. (31).

31 $$20\lg K = 12.29 \, \, {\text{dB}}$$

Then $K = 4.116$ can be solved, and if $T = 4500\pi$, the open-loop transfer function after adding the controller is:

32 $$H(s) = \left( {\frac{K}{Ts} + K} \right)G_{3}^{\prime} (s) = \frac{1.3019s + 18248.78}{{4.314 \times 10^{ - 5} s^{2} + s}}$$

**Figure 11:**
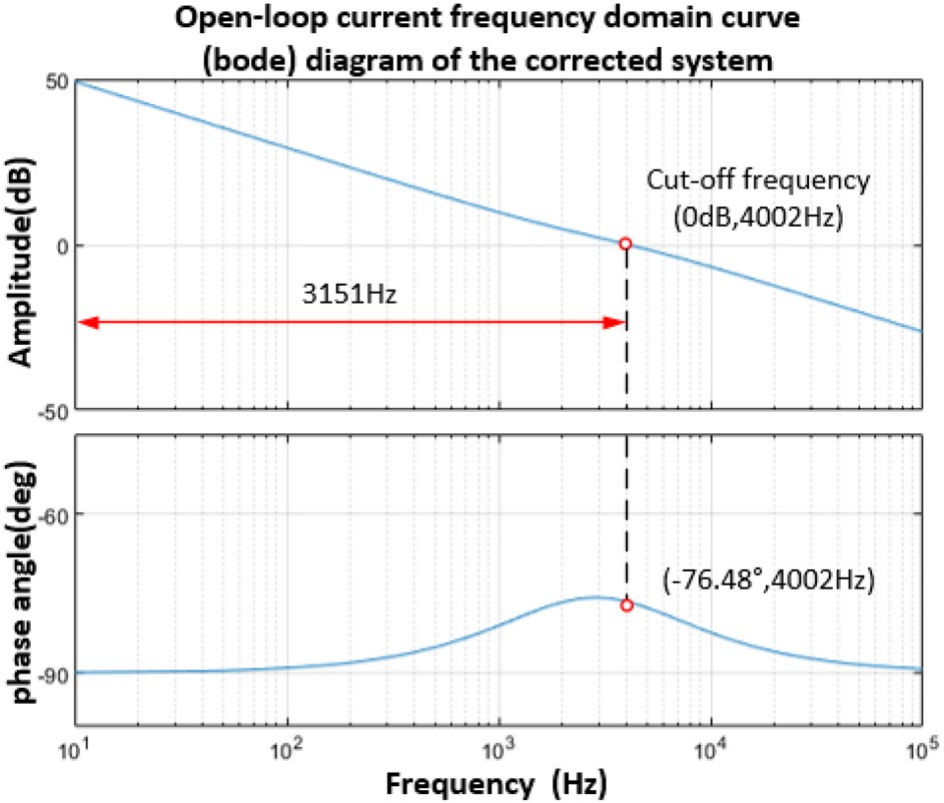
Bode diagram of current open-loop system.

**Figure 12:**
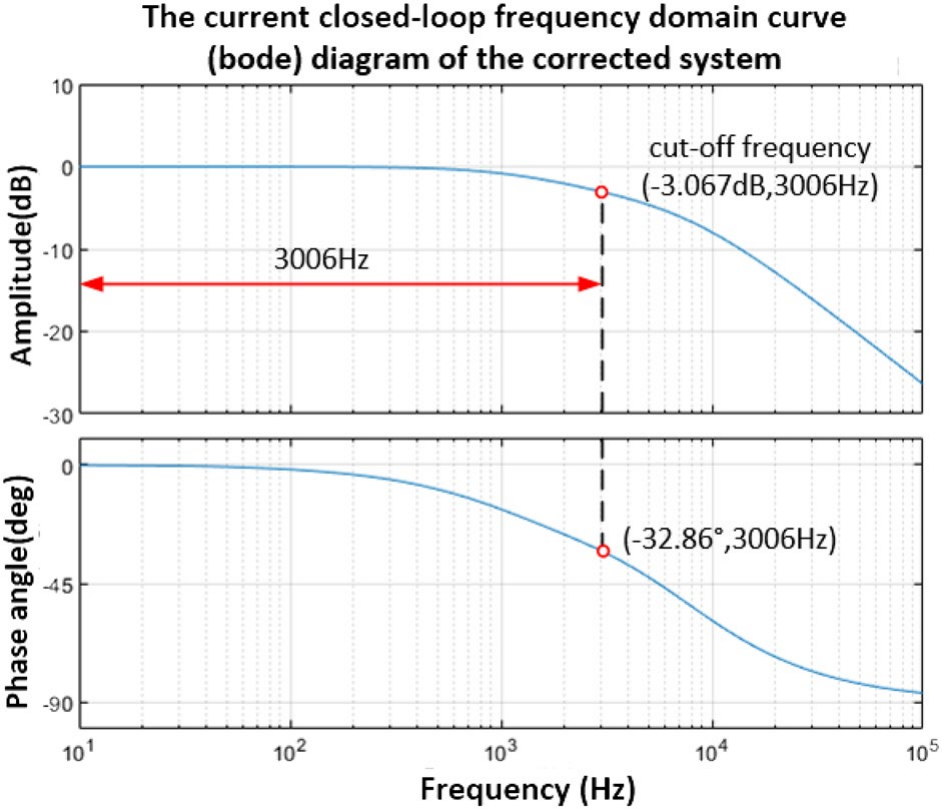
Bode diagram of current closed-loop system.

The Bode diagram obtained from Eq. (32) is shown in Fig. 11. The frequency corresponding to the point where the amplitude is 0 dB is 4002 Hz, the phase angle margin is 103.52°, and the amplitude margin is greater than 10 dB. The system is a stable system. Under the current loop control, the frequency domain characteristic curve of the closed-loop system is shown in Fig. 12, and the frequency corresponding to the point whose amplitude is − 3 dB is 3006 Hz.

#### Design of the controller of the vibration mirror velocity loop

**Figure 13:**
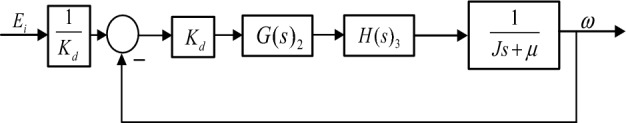
Block diagram of speed closed-loop system.

In order to explore the action mechanism of the speed loop controller, the current loop needs to be regarded as an intermediate link in the vibration mirror control system. Denoting the current loop as $H\left( s \right)_{3}$, the following closed-loop speed block diagram is obtained as shown in Fig. 13.

It is known that $H(s) = \frac{1.3019s + 18248.78}{{\left( {4.314 \times 10^{ - 5} } \right)s^{2} + s}}$, and the moment of inertia $J = 1.254 \times 10^{ - 7}$ kg m^2^, the friction damping coefficient $\mu = 5.19 \times 10^{ - 5}$, and $K_{d}$ is the feedback coefficient. The resulting velocity open-loop transfer function is shown below.

33 $$G(s)_{2} = \frac{{\left( {1.3019s + 18248.78} \right)K_{d} }}{{5.4098 \times 10^{ - 12} s^{3} + 1.2764 \times 10^{ - 7} s^{2} + 5.19 \times 10^{ - 5} s}}$$

The following form can be obtained by converting the Eq. (33) into the tail 1 canonical form.

34 $$G(s)_{2} = \frac{{\left( {2.508 \times 10^{4} s + 3.516 \times 10^{8} } \right)K_{d} }}{{s\left( {1.0424 \times 10^{ - 7} s^{2} + 2.459 \times 10^{ - 3} s + 1} \right)}}$$

Setting the feedback coefficient $K_{d} = 0.8$, the following open-loop transfer function is obtained as shown below.

35 $$G(s)_{2} = \frac{{2.0064 \times 10^{4} s + 2.813 \times 10^{8} }}{{s\left( {1.0424 \times 10^{ - 7} s^{2} + 2.459 \times 10^{ - 3} s + 1} \right)}}$$

**Figure 14:**
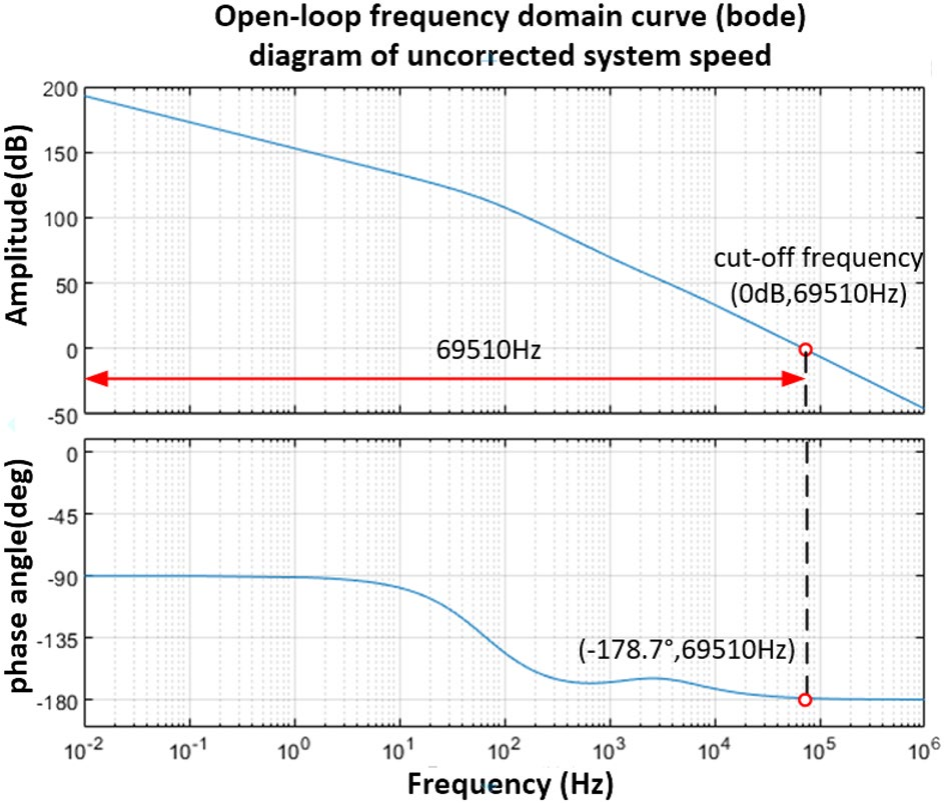
Open-loop transfer function of vibration mirror velocity loop.

It can be known from Eq. (35) that its open-loop Bode diagram is shown in Fig. 14. The amplitude margin and phase angle margin in the Fig. 14 are both positive, indicating that the system is stable. However, the phase angle margin of the system is 1.3°, and its stability is poor, so the phase angle margin of the system should be increased, thereby improving the stability of the system.

Set the phase angle margin $\gamma \ge 45^\circ$, the open loop cut-off frequency $\omega_{c} \ge 200{\text{Hz}}$, and the speed loop adopts the PD phase angle lead correction link.

As can be seen from the above Fig. 14$L(\omega_{c} ) = 96.92\;{\text{dB}}$, the PD controller transfer function is as follows.

36 $$G{(}s{)}_{2} = K_{v} \left( {{1} + \tau s} \right)$$

Let $s = j\omega$ be brought into (36) and simplified, the following relationship can be obtained.

37 $$G{(}j\omega {)}_{2} = K_{v} \left( {{1} + \tau j\omega } \right)$$

When $\mathop {{\text{lim}}}\limits_{\omega \to 0} K_{v} (1 + Tj\omega ) = K_{v}$, then $20{\text{lg}}\left| {\sqrt 2 K_{v} } \right| = - 95.61{\text{dB}}$, that is $K_{v} = 1.17217 \times 10^{ - 5}$.

Where $\tau = 400\pi$, and let $\omega_{c} = 200\;{\text{Hz}}$, the open-loop transfer function of the system after adding the PD controller is shown in Eq. (38).

38 $$G(s)_{2} = \frac{{2.1869 \times 10^{ - 4} s^{2} + 2.099135s + 3.2961 \times 10^{3} }}{{1.0424 \times 10^{ - 7} s^{3} + 2.459 \times 10^{ - 3} s^{2} + s}}$$

**Figure 15:**
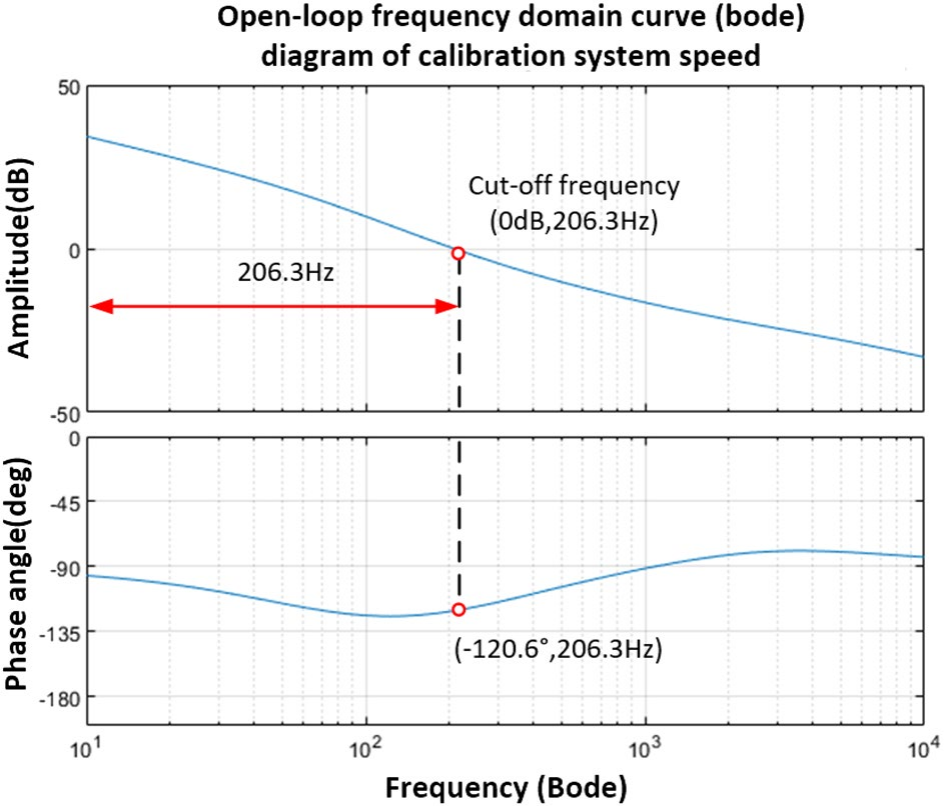
Bode diagram of speed open-loop system after PD controller correction.

After correction by the PD controller, the Bode of the vibration mirror velocity open-loop system is shown in Fig. 15.

It can be seen from Fig. 15 that the open-loop cut-off frequency is 206.3 Hz, the phase angle margin is 59.4°, and the amplitude margin also meets the system stability conditions.

**Figure 16:**
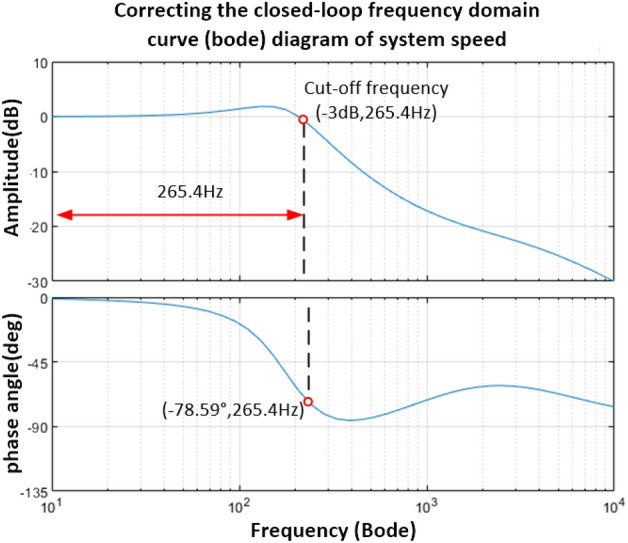
Bode diagram of speed closed-loop system after PD controller correction.

It is easy to obtain the speed closed-loop transfer function form as shown in Eq. (39), and its Bode diagram is shown in Fig. 16. The cut-off frequency corresponding to the − 3 dB point is 265.4 Hz.

39 $$H(s)_{2} = \frac{{2.1869 \times 10^{ - 4} s^{2} + 2.099135s + 3.2961 \times 10^{3} }}{{1.0424 \times 10^{ - 7} s^{3} + 2.67769 \times 10^{ - 3} s^{2} + 3.099135s + 3.2961 \times 10^{3} }}$$

#### Design of vibration mirror position loop controller

**Figure 17:**
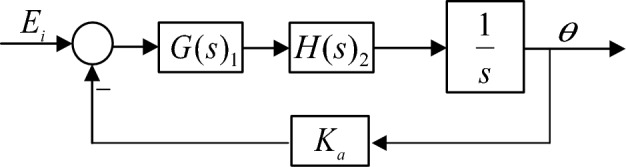
Block diagram of vibration mirror position closed-loop system.

The vibration mirror position loop is the outermost loop of the system, and the design of the position loop controller must first consider both the current loop and the velocity loop as intermediate links. The position loop loop can be simplified to obtain the block diagram form shown in Fig. 17.

In the Fig. 17, $H(s)_{2}$ is the current and speed loop transfer function, $G(s)_{1}$ is the position loop controller, and $K_{a}$ is the position feedback coefficient.

**Figure 18:**
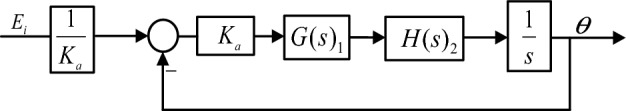
Block diagram of the vibration mirror position unit feedback system.

The closed-loop block diagram of the vibration mirror position can be further transformed into a unit feedback form, and the block diagram form shown in Fig. 18 can be obtained.

**Figure 19:**
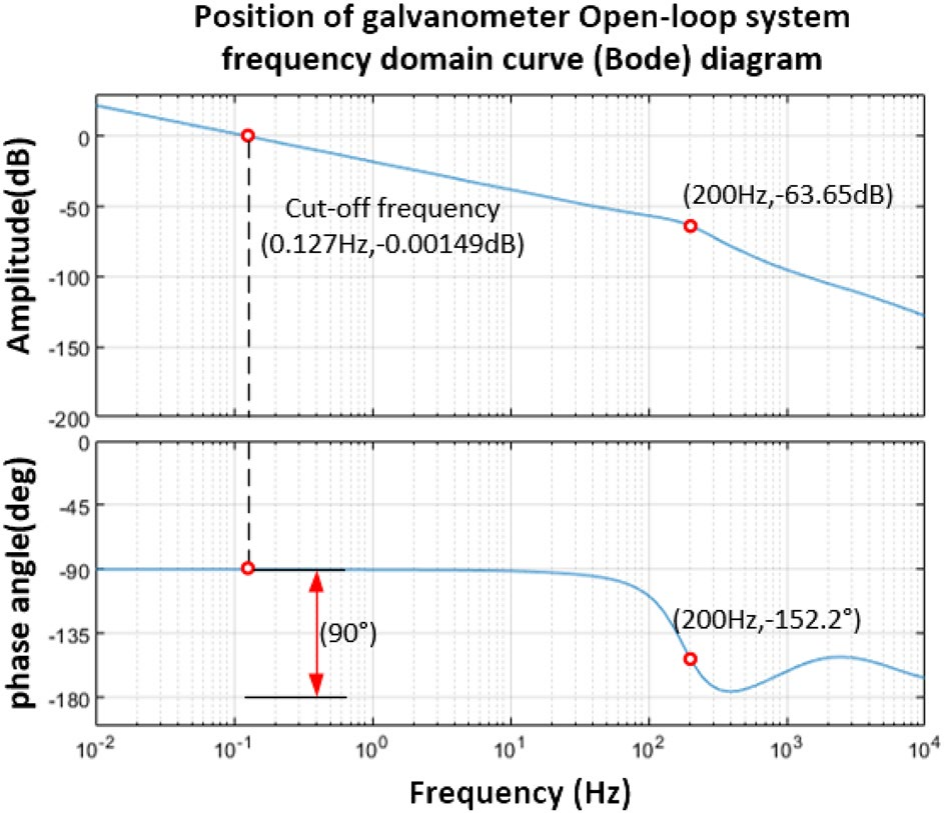
Bode diagram of vibration mirror position open-loop system.

According to the equation of the vibration mirror velocity loop in Eq. (39), it can be known that, if the position feedback coefficient is set $K_{a} = 0.8$, the open-loop transfer function form of the vibration mirror position loop is shown in Eq. (40), and the open-loop Bode diagram of the system is shown in Fig. 19:

40 $$G_{1} (s) = \frac{{0.8 \times \left( {2.1869 \times 10^{ - 4} s^{2} + 2.099135s + 3.2961 \times 10^{3} } \right)}}{{1.0424 \times 10^{ - 7} s^{4} + 2.67769 \times 10^{ - 3} s^{3} + 3.099135s^{2} + 3.2961 \times 10^{3} s}}$$

It can be seen from Fig. 19 that the crossover frequency is 0.127 Hz, the corresponding phase angle margin is 90°, and the amplitude margin meets the stability condition, but the value is small, indicating that the relative stability is poor. Therefore, it is necessary to adjust the system according to the requirements of the index, take $\omega = 200\;{\text{Hz}}$, the corresponding amplitude is $L(\omega ) = - 63.65\;{\text{dB}}$, and the phase angle is − 152.2°; its position loop selects the PD controller for correction.

41 $$20\lg \left| {K(1 + \tau s)} \right| = 63.65\;{\text{dB}}$$

Then $K = 1522.30$, set $\tau = 600\pi$ so the PD correction link is as follows.

42 $$G(s)_{1} = 1522.30\left( {1 + \left( {600\pi s} \right)} \right)$$

**Figure 20:**
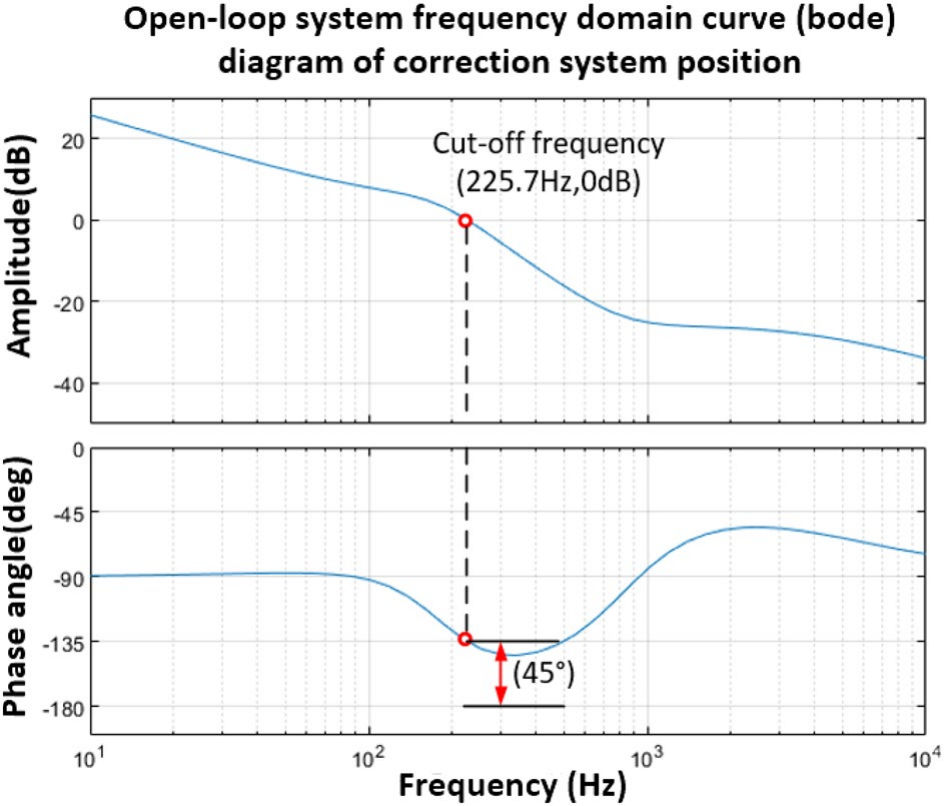
Bode diagram of position open loop system after PD controller correction.

Then the corrected open-loop transfer function is shown in Eq. (43), and its open-loop Bode diagram is shown in Fig. 20.

43 $$G_{1} (s)G(s)_{1} = \frac{{1.41292 \times 10^{ - 4} s^{3} + 0.99592s^{2} + 4.686 \times 10^{3} s + 4.0136 \times 10^{6} }}{{1.0424 \times 10^{ - 7} s^{4} + 2.67769 \times 10^{ - 3} s^{3} + 3.099135s^{2} + 3.2961 \times 10^{3} s}}$$

**Figure 21:**
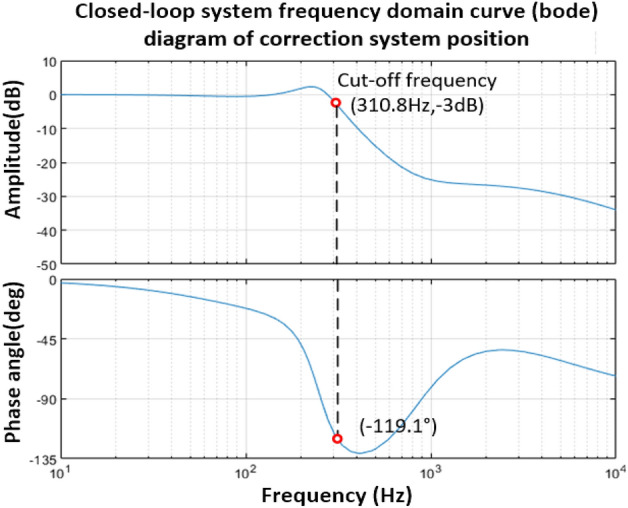
Bode diagram of the closed-loop system of the vibration mirror position after PD controller correction.

It can be seen from Fig. 20 that the corrected position open-loop amplitude margin satisfies the stability condition, and the phase angle margin is 45°. The closed-loop transfer function form of the vibration mirror position corrected by the PD controller is shown in Eq. (44). The Bode diagram of the system is shown in Fig. 21, and the bandwidth of the system is 310.8 Hz.

44 $$H{\prime} (s)_{1} = \frac{{1.41292 \times 10^{ - 4} s^{3} + 0.99592s^{2} + 4.686 \times 10^{3} s + 4.0136 \times 10^{6} }}{{1.0424 \times 10^{ - 7} s^{4} + 2.81898 \times 10^{ - 3} s^{3} + 4.09506s^{2} + 7.9821 \times 10^{3} s + 4.0136 \times 10^{6} }}$$

#### Simulation analysis of control system

Simulink function module under Matlab software is used to simulate and analyze the dynamic performance of the vibration mirror control system corrected by the controller. The 25–110 Hz sinusoidal signal is used as the input command, and the corresponding angular displacement output response is obtained. The mathematical model of this simulation takes into account the moment of inertia, viscous friction coefficient, and the influence of noise generated at the input signal in the working process.

**Figure 22:**
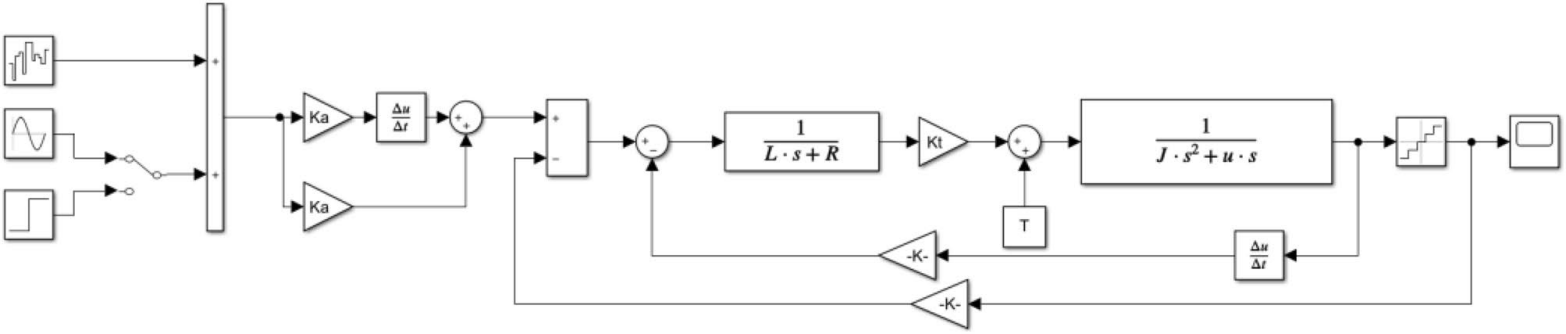
Simulink vibration mirror series correction system diagram.

**Figure 23:**
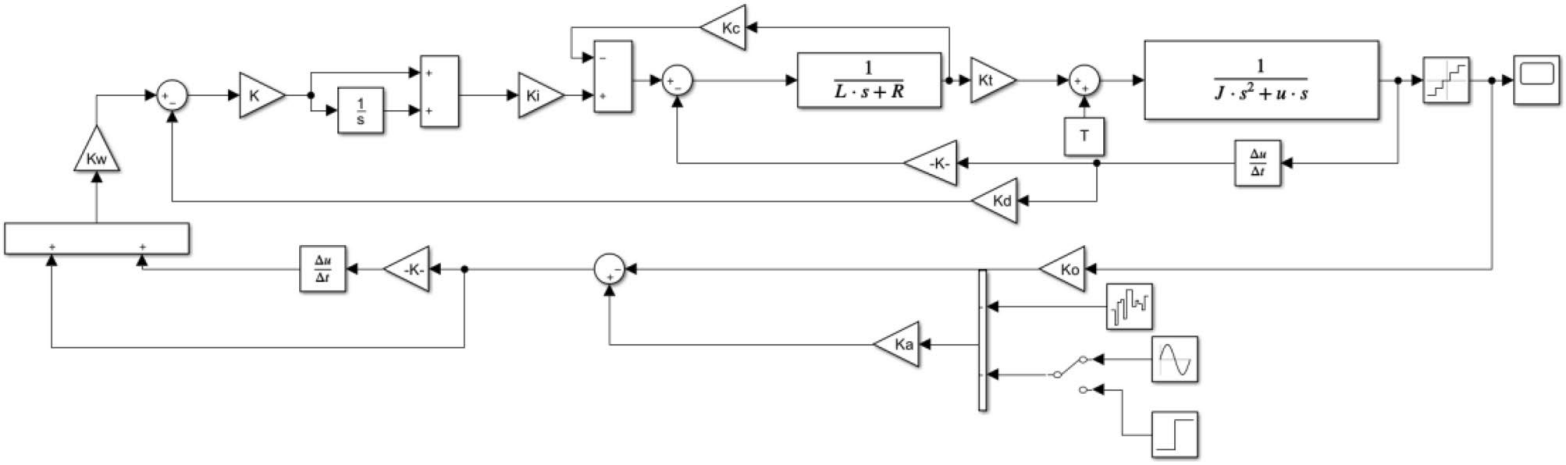
Simulink vibration mirror three closed-loop system controller diagram.

The three closed-loop system simulation models after series correction and current loop, speed loop and position loop synthesis are established by Simulink function module are shown in Figs. 22 and 23 respectively.

**Figure 24:**
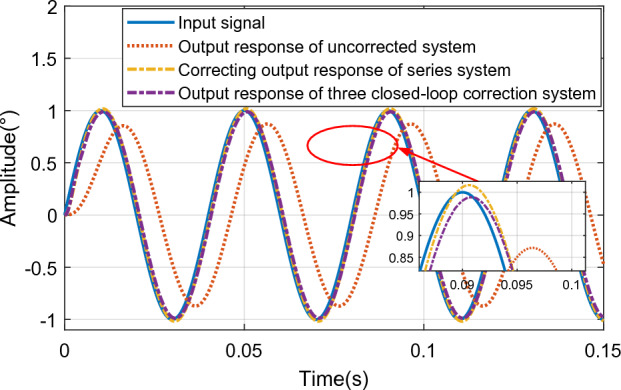
Output response curve of sinusoidal input 25 Hz system.

For the series correction system and the three closed-loop controller system respectively, a sinusoidal input signal with a frequency of 25 Hz and an amplitude of 1° is used, and the normally distributed random noise signal generated by the band-limited white noise block is mixed to observe the dynamic performance of the system, such as As shown in Fig. 24.

**Figure 25:**
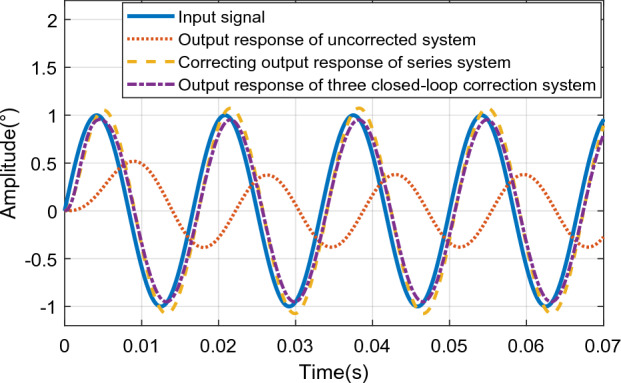
Output response curve of sinusoidal input 60 Hz system.

According to Fig. 24, when the input sinusoidal signal frequency is 25 Hz, the maximum error of the sinusoidal response of the system under the action of the series controller is 0.0165°, and the phase angle lag is 5.467°, that is, the system meets the double ten index requirements. The maximum value of the sinusoidal response amplitude error of the three-closed-loop control system is 0.0119°, which is less than 10% of the input command signal, and the phase angle lag is 7.193°, which meets the double ten index requirements of the control system. Through comparison, it can be seen that the steady-state accuracy of the system under the action of the three-closed-loop controller is higher, but the phase lag is slightly larger than that under the action of the series controller. Therefore, the control effect of the three-closed-loop controller is better than that of the series controller when the phase angle is not required. When the sinusoidal signal with input frequency of 60 Hz and amplitude of 1 is used as the input signal, the output response curve of the system is shown in Fig. 25.

As shown in Fig. 25, the maximum error of the sinusoidal response of the system under the action of the series controller is 0.074°, and the phase angle lag is 16.1°. The maximum error of the sinusoidal response of the system under triple closed-loop control is 0.048°, and its phase angle lags 15.28°. The output amplitudes of the system under series control and the system under three closed-loop control are both less than 10% of the input signal amplitude, which meets the requirements of the double ten index, but the steady-state error of the system under triple closed-loop control is smaller than that under series control.

**Figure 26:**
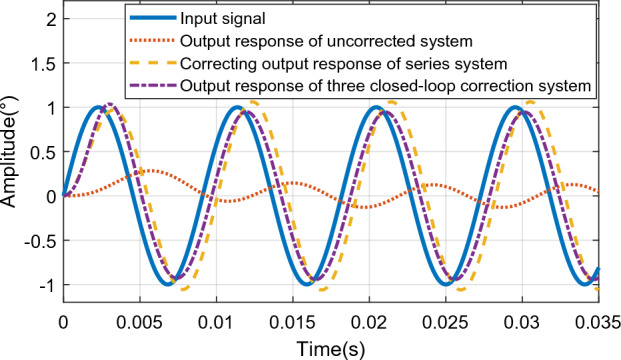
Output response curve of sinusoidal input 110 Hz system.

When a sinusoidal signal with an input frequency of 110 Hz and an amplitude of 1° is used as the input signal, the output response curve of the system is shown in Fig. 26.

As shown in Fig. 26, the maximum error of the sinusoidal response of the system under the action of the series controller is 0.061°, and the phase angle lag is 39°; the maximum error of the sinusoidal response of the system under the triple closed-loop control is 0.052°, and the phase angle lag is 23.64°; Through comparison, it can be seen that the three closed-loop control effect is still better than the series control effect, which meets the control index requirements of the actual system.

**Figure 27:**
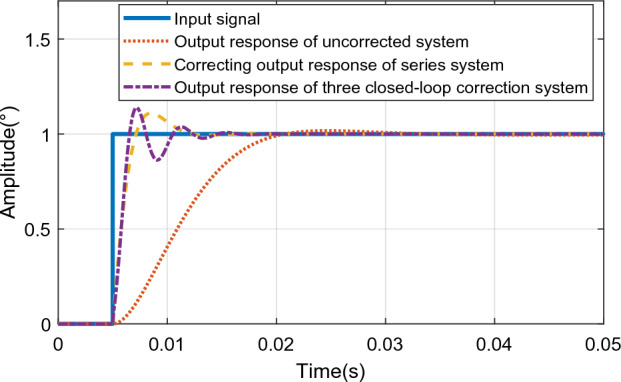
The output response curve of the unit step position with an amplitude of 0.1°.

When a unit step signal is input to the vibration mirror system, its output response is shown in Fig. 27.

The system step response index is shown in Table 2 below. It can be seen from Fig. 27 and Table 2 that the response speed of the vibration mirror system and the steady-state performance of the system are the best under the action of the three closed-loop controller.

**Table 2:** Dynamic performance indicators before and after system calibration.

	Overshoot	Risetime	Peak time	Adjusting time	Steady state error
No correction link	1.708%	*t*_*wc*_ = 0.022 s	*t*_*wp*_ = 0.204 s	*t*_*ws*_ = 0.013 s	*e*_*w*_ = 0.005
Tandem correction system	10.9%	*t*_*rc*_ = 0.0022 s	*t*_*pc*_ = 0.0026 s	*t*_*sc*_ = 0.0053 s	*e*_*c*_ = 0.003
Three closed-loop system	13.9%	*t*_r_ = 0.0016 s	*t*_*p*_ = 0.0018 s	*t*_*s*_ = 0.0029 s	*e*_*s*_ = 0

## Experimental verification of control strategy for galvanometer system

**Figure 28:**
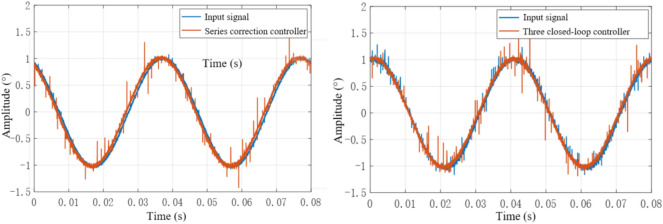
Output response curve of correction system for different controllers at 25 Hz.

By analyzing the mathematical model of the galvanometer system, we have designed a series and three closed-loop system calibration strategy, aiming to achieve high-frequency scanning of the galvanometer. This article uses Simulink simulation software to simulate and analyze the system after control strategy correction. The results show that the series correction strategy and three closed-loop correction strategy designed in the article can maintain good dynamic performance under high-frequency operating conditions. This chapter is based on the content of the previous chapter and verifies the applicability of the series and three closed-loop control strategies through experiments. Due to the more complex waveform of sine signals compared to step signals, it can more clearly describe the amplitude attenuation and phase lag characteristics of the system. Therefore, we selected sine signals of different frequencies as inputs and analyzed the dynamic performance of the system output. When the system inputs a sine signal with an amplitude of 1 V and a frequency of 25 Hz, the system output response corrected by different controllers is shown in Fig. 28. It can be observed that under low-frequency operating conditions, both series correction and three closed-loop correction controllers exhibit high dynamic accuracy, and there is no significant difference between them.

**Figure 29:**
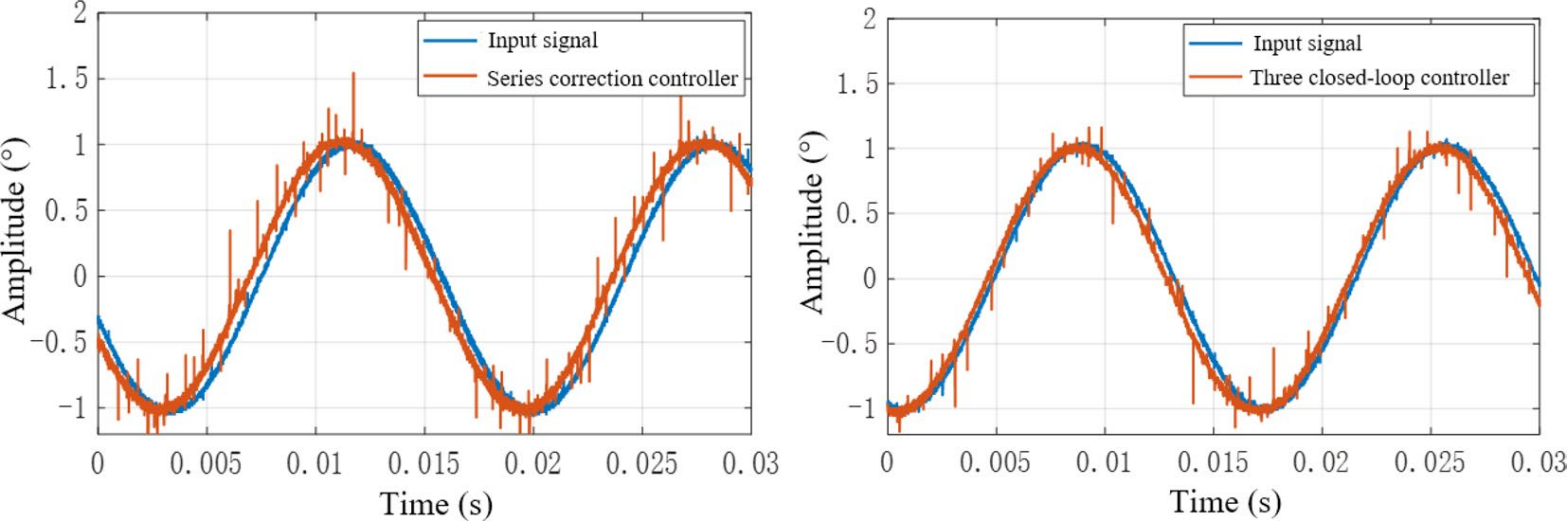
Output response curve of correction system for different 60 Hz controllers.

**Figure 30:**
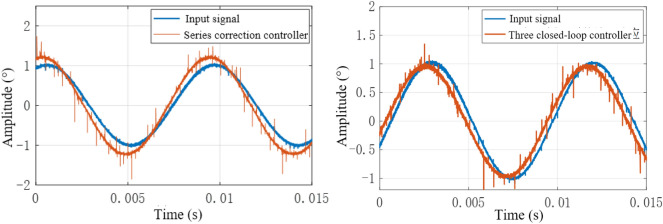
Output response curve of correction system for different controllers at 110 Hz.

When the system receives a sine signal with an amplitude of 1 V and a frequency of 60 Hz as input, the output signal of the system is shown in Fig. 29. As the frequency of the input signal increases, we observe a phase lag phenomenon between the input signal and the output signal, and the dynamic accuracy gradually decreases with the increase of frequency. When the frequency of the input sine signal of the system further increases to 110 Hz, the output signal of the system is shown in Fig. 30. From the figure, it can be seen that both the series correction system and the three closed-loop correction system exhibit amplitude attenuation and phase lag in their output response. However, it should be pointed out that the system after three closed-loop correction performs better in terms of dynamic accuracy compared to the series correction system. Therefore, the variation pattern is relatively close to the simulation results.

This section conducts dynamic performance tests on systems calibrated by different controllers. The results show that the classic control strategy enables the system to have small dynamic and static errors during medium to low frequency operation. However, as the frequency increases, there are phenomena of amplitude attenuation and phase lag. It is worth noting that there is some consistency between the experimental results and the simulation results. In the low-frequency range, the experimental results are in good agreement with the simulation results, while in high-frequency operation, the difference is within 10%. This indicates that the designed controller has quite reasonable performance in different frequency ranges.

## Conclusion

In this paper, the working principle of vibration mirror system is analyzed, and the mathematical model of each component is established, and then the mathematical model and simulation model of the whole vibration mirror system are obtained. By studying the frequency domain curve and time domain curve of vibration mirror system, the main conclusions are as follows:

1. The viscous friction coefficient of the vibration mirror system has a great influence on the frequency domain characteristics and time domain response of the system. The study found that the viscous friction coefficient will make the system show different damping characteristics, the larger the viscous friction coefficient, the greater the damping of the system. Therefore, when studying the vibration mirror control system, the viscous friction of the system should be taken into account.
2. Under the action of the series correction controller and the triple closed-loop controller, the overshoot of the system step response is 10.9% and 13.9%, respectively. From the perspective of system speed, accuracy, and stability, the three closed-loop correction system exhibits superior dynamic performance compared to the series correction system. It can effectively eliminate steady-state errors and has faster system response speed.
3. The research results indicate that under the stimulation of sinusoidal signals, both the series correction system and the three closed-loop correction system exhibit an increasing phase lag phenomenon with increasing frequency. However, the output response amplitude error of the two systems is relatively small, which can meet the working requirements. The comparative analysis of experimental and simulation results shows that the difference between the two systems is within the range of 10%. Therefore, the control strategy proposed in this article can achieve excellent dynamic and static performance, suppress interference, and reduce steady-state error of the galvanometer system under high-frequency signal excitation. Compared to intelligent control strategies and artificial neural network control methods, this control method exhibits significant advantages in terms of economy and complexity, while helping engineers and operators more easily adjust and optimize the control system.

## Data Availability

The datasets used and/or analysed during the current study available from the corresponding author on reasonable request.
